# Y-box Protein-1 Regulates the Expression of Collagen I in Hepatic Progenitor Cells via PDGFR-*β*/ERK/p90RSK Signalling

**DOI:** 10.1155/2017/6193106

**Published:** 2017-08-27

**Authors:** Fei Li, Zhenzeng Ma, Heng Liu, Qidi Zhang, Xiaobo Cai, Ying Qu, Mingyi Xu, Lungen Lu

**Affiliations:** ^1^Department of Gastroenterology and Hepatology, Shanghai General Hospital, Shanghai Jiao Tong University School of Medicine, Shanghai 200080, China; ^2^Gastroenterology Department, The First Affiliated Hospital of Anhui Medical University, Hefei, Anhui 230022, China

## Abstract

Y-box protein-1 (YB-1) is a highly conserved transcription factor that is involved in multiple biological processes via transcriptional regulation of several genes, including *p53*, *cyclin D1*, and *EGFR*. YB-1 has been reported to be overexpressed in injured livers. This study aims to explore the functions of YB-1 in hepatic progenitor cells (HPCs). Herein, chromatin immunoprecipitation sequencing (ChIP-sequencing) and RNA-sequencing assays identified that YB-1 participated in the biological adhesion process and ECM-receptor interactions in HPCs. Further study demonstrated that YB-1 modulated the expression of extracellular matrix components in HPCs. ChIP-sequencing assays established that *PDGFR-β* was a target gene of YB-1, and luciferase reporter assays confirmed that YB-1 negatively regulated *PDGFR-β* promoter activity in HPCs. In addition, PDGFR-*β* can regulate the expression of collagen I through ERK/p90RSK signalling, and disruption of the signalling pathway with a PDGFR-*β* inhibitor or ERK1/2 inhibitor abolished the regulatory effect of PDGFR-*β* on collagen I expression in HPCs. Conclusively, YB-1 can modulate the expression of collagen I in HPCs via direct binding to the *PDGFR-β* promoter, negatively regulating its expression. In addition, the ERK/p90RSK axis serves as the downstream signalling pathway of PDGFR-*β*.

## 1. Introduction

YB-1 belongs to a family of DNA- and RNA-binding factors, also named cold shock proteins, which are highly conserved during evolution and have been shown to function as regulators of gene transcription and translation. A wide range of nucleic acid structures are reported to be specifically bound to YB-1, most of which harbour an inverted CCAAT-box (ATTGG) as the core binding site. YB-1 was first recognized as a protein that binds to the promoter of the major histocompatibility complex II gene HLA-DR*α* and that negatively regulates the expression of this gene [1]. Later, a large number of studies regarding the functions of YB-1 were conducted, and they demonstrated multiple effects of YB-1 on cell proliferation, migration, and transformation. YB-1 directly interacts with *p53*, and YB-1 knockdown upregulates endogenous *p53* and induces various tumour cell lines to undergo apoptosis [2, 3]. Meanwhile, YB-1 has been found to bind to a number of genes, including *cyclin D1*, epithermal growth factor receptor (*EGFR*), and mitogen-activated protein kinase-interacting kinase 1 (*MNK1*), and then modulate the transcription of these genes and the proliferation rate of related tumour cells [4–6]. Furthermore, YB-1 also participates in the production of the extracellular matrix (ECM) and in the scarring process. In human embryonic kidney cells and dermal fibroblasts, YB-1 exerted a repressive effect on the *collagen α1 (I) (COL1A1)* and *matrix metalloproteinase-2 (MMP-2)* gene promoters [7, 8].

YB-1 has been reported to be involved in liver development and liver diseases. Grant and his colleagues found that YB-1 was relatively abundant in the liver at day 7 of embryogenesis and decreased steadily throughout chicken embryogenesis. In addition, they found that YB-1 mRNA in rat livers was elevated approximately 10- and 6-fold 24 and 48 h after administration of carbon tetrachloride, respectively, which was accompanied by DNA synthesis and cell proliferation [9]. Gunasekaran et al. reported that most hepatocellular carcinoma tissues expressed YB-1 and showed a relatively higher expression compared to normal livers [10]. In addition, in vitro experiments demonstrated that YB-1 was a potent inducer of Smad7 expression in activated hepatic stellate cells (HSCs) and myofibroblastic mesangial cells, which could be used to antagonize TGF-*β* in chronic stages of fibroproliferative diseases in the liver [11]. In an inflamed liver, YB-1 suppressed the synthesis of collagen and modulated fibrogenesis [12].

Based on the above studies, we speculate that YB-1 may play certain roles in liver regeneration and fibrogenesis. In the present study, we attempted to determine the effects of YB-1 on hepatic progenitor cells, as well as the possible molecular mechanism. The results would help booster our understanding of liver repair and fibrogenesis.

## 2. Materials and Methods

### 2.1. Animals

C57BL/6J mice (4 weeks old) were purchased from the Sino-British Sippr/BK Laboratory and housed in the Animal Experimental Center of Shanghai First People's Hospital (Shanghai, China) under specific pathogen-free conditions. The Chancellor's Animal Research Committee approved all the animal studies and confirmed that the experiments involving animals adhered to the guidelines set forth by the Shanghai Jiao Tong University School of Medicine (Shanghai, China).

### 2.2. Isolation of HPCs and Cell Culture

Four-week-old, male, wild-type C57BL/6J mice were fed a 50% choline-deficient diet (Trophic, Nantong, China) plus 0.15% ethionine solution in drinking water (CDE diet). After 3 weeks on the diet, mice were anaesthetised by intraperitoneal Nembutal injection (1.5% Pelltobarbitaium Natricum, Sigma-Aldrich; 0.1 ml/20 g body weight). HPCs were then isolated using a modified two-step perfusion protocol as previously described [13]. The cells were seeded in 90 mm culture dishes and cultured in complete William's E medium supplemented with 10% FBS, 2 mM glutamine, 100 U/ml antibiotics, 20 ng/ml epidermal growth factor (EGF, Peprotech), 30 ng/ml human insulin-like growth factor II (IGF-II, GroPep), and 10 *μ*g/ml insulin (Gibco). A week later, clones were selected by local trypsinization in clonal rings, and cells were resuspended in complete William's E medium. After culturing for 3 generations, purified HPCs were obtained and used for the following experiments.

For most experiments, HPCs were cultured in complete medium. In the indicated experiments, to detect the capacity of HPCs to secrete PDGF-*β*, FBS-free William's E medium was used. To analyse PDGFR-*β* signalling, the PDGFR-*β* inhibitor DMPQ (Abcam, Cambridge, UK) and ERK inhibitor FR180204 (Selleck, Houston, USA) were added to the medium.

### 2.3. Construction of YB-1 shRNA Lentiviruses and RNA Interference

The lentiviral vector LV-3 (GenePharma, Shanghai, China) containing a GFP reporter was used to express shRNA targeting the sequence of YB-1 (#1: 5′-GAGAGCAAGGTAGACCAGTGA-3′ and #2: 5′-GTCAAATGGTTCAATGTAAGG-3′) and scramble control (5′-TTCTCCGAACGTGTCACGT-3′). Briefly, LV-3-shYB-1 plasmids were transfected into HEK 293T cells with packaging vectors. The supernatant containing infective lentiviruses was collected 72 h posttransfection, and the lentiviruses were concentrated by ultracentrifugation for 2 h at 100,000 ×g and resuspended in PBS. HPCs were seeded in a 24-well plate well and infected with lentiviruses in the presence of 5 *μ*g/mL of polybrene. YB-1-knockdown cells were screened with 2 *μ*g/mL puromycin for 15 days postinfection.

### 2.4. Chromatin Immunoprecipitation Assays

Preparation of chromatin and chromatin immunoprecipitation (ChIP) assays were performed using a ChIP assay kit (Upstate, NY, USA) according to the kit protocol. Chromatin equal to 2 × 10^6^ cells was used for each immunoprecipitation. Chromatin immunoprecipitations were performed using HPC chromatin with normal rabbit IgG (negative control), anti-RNA polymerase II (positive control), and anti-YB-1 antibody (Abcam, ab76149). Precleared chromatin was immunoselected, processed, and subjected to PCR amplification with platinum Taq polymerase (Invitrogen, Carlsbad, CA) using sequence-specific PCR primers to amplify the region of the PDGFR-*β* promoter containing the YB-1 binding site. For positive control, primers specific for the GAPDH promoter containing the RNA polymerase II binding site were applied. Detailed information regarding the primers is listed in Table 1. Reaction products were subjected to Tris/agar gel electrophoresis. Gels were stained for 5 min in 1 *μ*g/ml ethidium bromide (BioRad, Hercules, CA) and visualized using a ChemiScope 2850 imaging system (CLiNX, Shanghai, China).

For chromatin immunoprecipitation sequencing (ChIP sequencing) assays, 1 × 10^7^ cells were used for each immunoprecipitation. ChIP sequencing was performed using an Illumina HiSeq2500 platform. Subsequent peak calling and motif analysis were conducted using HOMER, a software suite for ChIP-seq analysis.

### 2.5. Plasmid Construct, Transient Transfection, and Dual-Luciferase Assay

The pGL4-PDGFR-*β*-luciferase promoter reporter construct contained a *PDGFR-β* promoter sequence spanning base pairs −1322 to +173 from the transcription start site ligated upstream of the luciferase gene in the pGL4 vector (Promega, Fitchburg, USA).

YB-1 knockdown or scramble control HPCs were cotransfected with the pGL4-PDGFR-*β* constructs and pRL-CMV Renilla luciferase control reporter constructs using Lipofectamine 3000 reagent (Invitrogen, Carlsbad, CA) according to the manufacturer's protocol. Following 48 h of incubation, the cells were rinsed twice with PBS, and dual-luciferase assays were performed according to the manufacturer's protocol (Promega). Transcriptional activities of reporter constructs were normalized against those of cotransfected pRL-CMV Renilla luciferase control reporter vectors (Promega, Fitchburg, USA).

### 2.6. Western Blotting Analysis

Whole-cell lysates were prepared from culture cells. Medium supernatant protein was obtained by ultracentrifugation using Amicon Ultra-15 10K centrifugal filter devices (Millipore, MA, USA). Cellular or supernatant protein (20 *μ*g per well) was loaded into 10% acrylamide gels, and then, electrophoresed proteins were transferred to PVDF membranes, and membranes were blocked for 30 min in TBST containing 5% BSA. Primary antibodies were incubated for 12 h at 4°C and washed three times with TBST, followed by 2 h of incubation with specific IgG-HRP used at 1 in 6000 dilution in 1% BSA-TBST. After washing, bands were detected with an ECL system.

### 2.7. RNA Extraction, RT-PCR, and RNA Sequencing

Total RNA was isolated using TRIzol reagent (Invitrogen). Reverse transcription was performed using PrimeScript™ RT Master Mix (Takara, Dalian, China). All primer sets for PCR are listed in Table 2. PCR was performed using SYBR® Premix Ex Taq™ (Takara, Dalian, China) under the following conditions: 1 cycle at 95°C for 2 min, 35 cycles at 95°C for 15 sec and at 59°C for 34 sec, and 1 min at 72°C. The relative mRNA expression levels were normalized against those of the GAPDH gene in the same RNA preparation.

For RNA sequencing, RNA isolation was performed using Qiagen RNEasy Mini Kit (Qiagen, Hilden, Germany) according to the protocol provided by the manufacturer, and RNA sequencing was performed using an Illumina HiSeq2500 platform.

### 2.8. Immunofluorescence Staining and Confocal Microscopy

The liver tissue was fixed in formalin and embedded in paraffin. Then, 4 *μ*m specimens were prepared. After antigen retrieval with Tris-EDTA (pH 9.0) for 10 min at 95°C, the specimens were blocked with 5% bovine serum albumin (BSA) for 30 min at room temperature. The primary antibodies were applied overnight at 4°C in 1% BSA. Information regarding the primary antibodies is listed in Table 3. The secondary, fluorescently labelled antibodies were applied for 1 h at room temperature. Sections were counterstained with 4′,6-diamidino-2-phenylindole (DAPI). Fluorescence images were acquired with a Leica TCS microscope.

For cell labelling, cells were cultured on 0.2% poly-L-lysine-coated glass coverslips. After fixation in 4% paraformaldehyde for 20 min at room temperature, cells were permeabilized in PBS containing 0.1% Triton-100 and 5% BSA and incubated with specific primary antibodies overnight at 4°C. Then, the cells were stained with specific, fluorescently labelled secondary antibodies for 1 h at room temperature. After staining with DAPI and washes, confocal laser scanning microscopy and double immunofluorescence assays were performed using a Leica TCS microscope.

### 2.9. Gene Ontology (GO) Analysis

GO analysis was performed to analyse the main function of the DEGs according to gene ontology which is the key functional classification of NCBI. Fisher's exact test and *χ*^2^ test were used to classify the GO category, and the false discovery rate (FDR) was calculated to correct the *P* value; the smaller the FDR, the smaller the error was in judging the *P* value. The significant GO terms were defined with a *P* value of 0.05 and an FDR < 0.05.

### 2.10. Pathway Analysis

Pathway analysis was used to determine the significant pathways of the DEGs according to KEGG. Similarly, we used Fisher's exact test and *χ*^2^ test to select the significant pathways, and the threshold of significance was defined by *P* value and FDR. The significant pathways were identified with a *P* value < 0.05 and an FDR < 0.05.

### 2.11. Statistical Analysis

The data represent the mean ± SE. Statistical significance was assessed using one-way ANOVA and *t*-tests, and analysis was facilitated by the GraphPad InStat software program version 3.0.

## 3. Results

### 3.1. Defining the YB-1 Cistrome in HPCs

**Figure 1:**
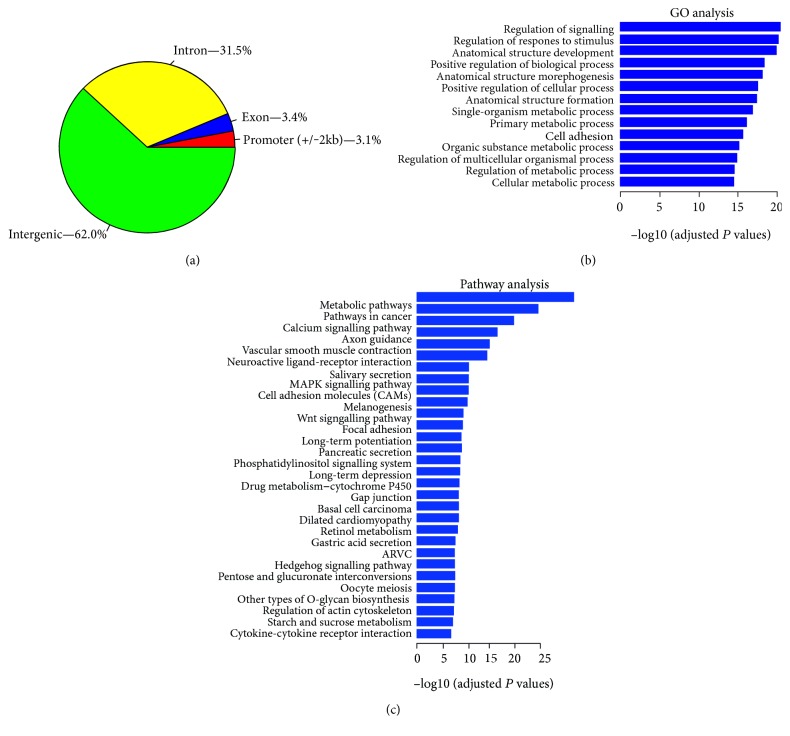
The YB-1 cistrome in hepatic progenitor cells. (a) Pie chart illustrating genomic locations of YB-1 binding sites in hepatic progenitor cells. Promoter regions, ±2 kb from the TSS. (b) Gene ontology (GO) analysis of genes annotated with YB-1 binding sites. (c) Pathway analysis of genes annotated with YB-1 binding sites.

To clarify the biological functions of YB-1, we analysed the genome-wide binding sites of YB-1 in HPCs using chromatin immunoprecipitation coupled with high-throughput deep sequencing (ChIP sequencing). The resulting cistrome identified 8524 binding sites. The majority of binding sites were localized to distant intergenic and intronic regions, 3609 (62%) and 1821 (31.5%), respectively. The rest bind to the exon (202, 3.4%) and promoter regions (192, 3.1%), as shown in Figure 1(a) and Supplementary Table 1 available online at https://doi.org/10.1155/2017/6193106. Gene ontology (GO) analysis of these annotated genes revealed that the most common functions for YB-1 target genes were regulation of signalling and regulation of response to stimulus. In addition, these target genes participate in cell adhesion (Figure 1(b)). Pathway analysis based on KEGG indicated that YB-1 target genes involve a variety of extremely important pathways, including metabolic pathways, the MAPK signalling pathway, cell adhesion molecules, the wnt signalling pathway, focal adhesions, and the hedgehog signalling pathway (Figure 1(c)).

### 3.2. Genome-Wide Expression Profiling of HPCs after YB-1 Knockdown

**Figure 2:**
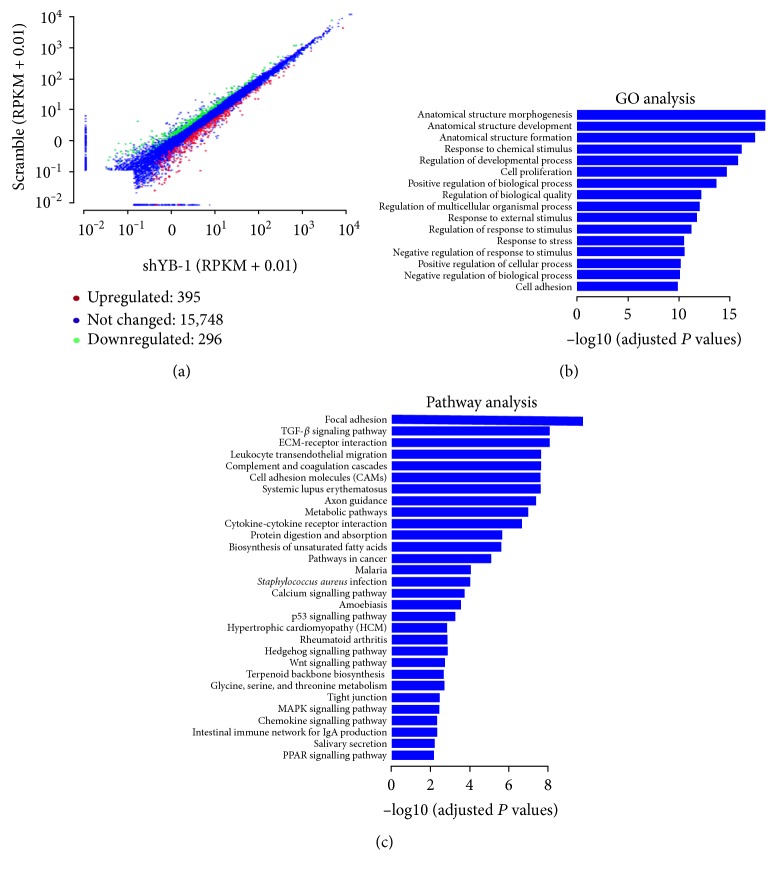
Differentially expressed genes (DEGs) of HPCs with YB-1 knockdown. (a) Overview of DEGs of HPCs with YB-1 knockdown. Log2-fold change > 0.585 or Log2-fold change < −0.585, and *P* value < 0.05. (b) Gene ontology (GO) analysis of DEGs. (c) Pathways enrichment analysis of DEGs based on KEGG.

To further determine the effects of YB-1 in HPCs, RNA sequencing was performed to screen the differentially expressed genes (DEGs) between YB-1 knockdown HPCs and the scramble control using the following criteria: log2-fold change > 0.585 or log2-fold change < −0.585, and *P* value < 0.05. Overall, we detected 691 DEGs between YB-1 knockdown HPCs and the scramble control, as shown in Supplementary Table 2. Among these, 395 genes were upregulated in the YB-1 knockdown HPCs and 296 genes were downregulated (Figure 2(a)). Additionally, we performed GO analysis and pathway analysis of the DEGs. Specifically, DEGs were associated with multiple GO terms, including anatomical structure morphogenesis, anatomical structure development, cell proliferation, and cell adhesion (Figure 2(b)). Pathway analysis revealed that the DEGs were involved in focal adhesions, the TGF-beta signalling pathway, ECM-receptor interaction, cell adhesion molecules, the hedgehog signalling pathway, and the wnt signalling pathway (Figure 2(c)). In addition, we found that a number of DEGs involved in ECM-receptor interaction processes were upregulated, as listed in Table 4.

### 3.3. YB-1 Modulates Synthesis of the Extracellular Matrix (ECM) in HPCs

**Figure 3:**
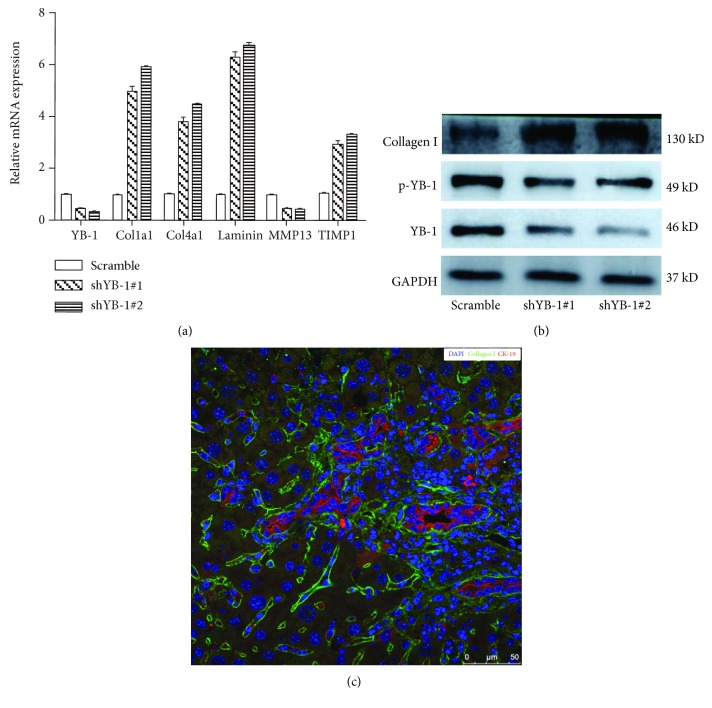
YB-1 regulates the synthesis of ECM proteins. (a) YB-1 knockdown promoted the mRNA expression of Col1a1, Col4a1, laminin, and TIMP-1, but suppressed the expression of MMP13. (b) Western blot assays provided evidence that YB-1 knockdown enhanced collagen I expression in HPCs. (c) CDE-injured mouse liver sections were stained with CK-19 and collagen I, and the results showed that collagen I can be expressed by a number of single dispersed CK-19-positive cells, which represented a subpopulation of HPCs.

Because ECM components, including collagens and laminin, are the major participants in cell adhesion, we examined if HPCs express the components involved in metabolism of the ECM. Surprisingly, we found that HPCs expressed collagen I, collagen IV, and laminin as well as the related metabolic enzymes (Figure 3(a)). Moreover, YB-1 knockdown with a lentiviral vector elevated the expression of collagen I, collagen IV, and laminin. In addition, YB-1 knockdown suppressed matrix metalloproteinase 13 (MMP13) and promoted the expression of tissue inhibitor of metalloprotease 1 (TIMP-1) at the mRNA level (Figure 3(a)). Western blot assays also demonstrated that knockdown of YB-1 was accompanied by decreased expression of the active phosphorylated YB-1 structure and overexpression of collagen I (Figure 3(b)). Immunofluorescence staining revealed that dispersed CK-19-positive HPCs produced collagen I (Figure 3(c)). These findings suggest that HPCs have the capacity to produce ECM and that YB-1 can negatively modulate this process.

### 3.4. YB-1 Negatively Regulates PDGFR-*β* Transcriptionally

**Figure 4:**
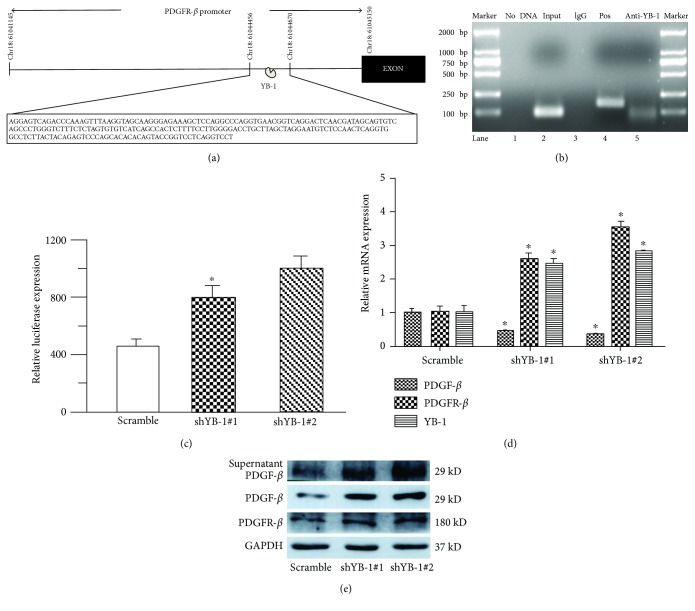
YB-1 directly binds to the PDGFR-*β* promoter and negatively regulates its expression. (a) ChIP-sequencing assays detected that YB-1 directly bound to a region of the PDGFR-*β* promoter spanning from the site 61044456 to 61044670 on chromatin 18. (b) ChIP-PCR confirmed that PDGFR-*β* is one of the YB-1 target genes. Chromatin immunoprecipitations were performed using HPC chromatin with normal rabbit IgG, anti-RNA polymerase II, and anti-YB-1 antibodies. Purified DNA was then analysed with PCR using primers specific for YB-1 binding sites on the PDGFR-*β* promoter or primers for the GAPDH promoter as the positive control. The size of the PCR product for the PDGFR-*β* promoter and the GAPDH promoter was 82 and 166 bp, respectively. The PCR product was observed in the input, positive, and anti-YB-1 ChIPs (lanes 2, 4, and 5) but not in the no DNA and IgG ChIPs (lanes 1 and 3). (c) YB-1 negatively modulates PDGFR-*β* promoter activity in HPCs. HPCs were transfected with a scramble lentivirus or YB-1 knockdown lentivirus; PDGFR-*β* promoter-luciferase constructs and Renilla luciferase constructs were cotransfected into HPCs. Cells were harvested 48 h after the second transfection, and luciferase activity was measured. The results are an average of three independent transfections. (d) YB-1 knockdown enhanced PDGFR-*β* mRNA expression. In addition, PDGF-*β* expression was elevated in HPCs with YB-1 silenced. (e) Western blotting revealed that YB-1 knockdown negatively regulated PDGFR-*β* and PDGF-*β* expression. Compared with the culture supernatant from HPCs transfected with the scrambled lentivirus, the YB-1 knockdown groups showed higher levels of PDGF-*β* protein. ^∗^*P* < 0.01, compared with the scramble control.

To investigate the detailed mechanism by which YB-1 regulates the expression of ECM components, ChIP sequencing and RNA sequencing were introduced to identify the potential target of YB-1. The ChIP-sequencing approach suggested that YB-1 directly bound to the promoter region of *PDGFR-β* ranging from the site 61044456 to 61044670 on chromatin 18, −640 bp from the transcription start site, and ChIP-PCR confirmed that the PCR product is of the expected size, as shown in Figures 4(a) and 4(b). In addition, RNA sequencing showed that PDGFR-*β* mRNA was elevated in the YB-1 knockdown HPCs compared to the scramble HPCs (see Supplementary Table 2). In addition, a variety of studies in the literature have reported that PDGFR-*β* is upregulated in injured livers and contributes to liver cirrhosis [14, 15]. Therefore, we chose to pursue PDGFR-*β* as a candidate to reveal the molecular mechanism by which YB-1 regulates ECM homeostasis. Next, luciferase reporter assays were used to determine whether YB-1 affected transcription of the *PDGFR-β* promoter. Reporter constructs containing the *PDGFR-β* promoter spanning the site −1322 to +173 cloned upstream of the luciferase gene were transiently transfected into the scramble HPCs and YB-1 knockdown HPCs. The data suggested that YB-1 knockdown promoted *PDGFR-β* promoter activity (Figure 4(c)). We also demonstrated that YB-1 silencing increased the expression of PDGFR-*β* and its ligand PDGF-*β* both in the cytoplasm and the culture medium (Figures 4(d) and 4(e)).

### 3.5. YB-1 Affects Collagen I Expression via PDGFR-*β*/ERK/p90RSK Signalling

**Figure 5:**
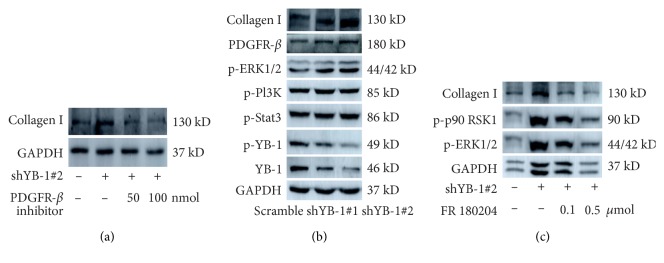
YB-1 regulates collagen I expression via PDGFR-*β*/ERK/p90RSK signalling. (a) YB-1 silencing enhanced collagen I expression in HPCs, but the PDGFR-*β* inhibitor DMPQ blunted the regulatory effect of YB-1 silencing on collagen I expression. (b) Seven days after YB-1 knockdown, HPCs were harvested to analyse the signalling pathway involved in YB-1-regulated collagen I expression. Following YB-1 silencing, phosphorylated YB-1 also decreased, while PDGFR-*β* and phosphorylated ERK1/2 were elevated. The level of phosphorylated Stat3 and PI3K showed no alteration. (c) The ERK1/2 inhibitor FR180204 repressed phosphorylation of p90RSK1 and ERK1/2 and abolished YB-1-regulated collagen I expression.

Because YB-1 negatively regulates both collagen I and PDGFR-*β*, we sought to investigate whether PDGFR-*β* could modulate the expression of collagen I. In this study, the PDGFR-*β* inhibitor DMPQ was utilized to block the PDGFR-*β* signalling pathway. Surprisingly, the effect of YB-1 knockdown on the production of collagen I was abolished by the PDGFR-*β* inhibitor in a dose-dependent manner (Figure 5(a)). Meanwhile, we detected the key components of PDGFR-*β*-related signalling pathways that may participate in the expression of collagen I in HPCs. Western blotting assays showed that phosphorylated ERK1/2 but not phosphorylated PI3K or Stat3 mediated PDGFR-*β*-induced collagen I production (Figure 5(b)). Further studies demonstrated that phosphorylation of p90 RSK at Ser380 synchronized with the amount of phosphorylated ERK1/2 and collagen I (Figure 5(c)).

## 4. Discussion

YB-1 has been reported to be elevated in HCC tissues and in regenerating liver after injury [9, 10]. In this study, ChIP sequencing and RNA sequencing provided clues that YB-1 participated in the biological adhesion process and ECM-receptor interaction in HPCs. Therefore, we detected the ligand components, including collagen and laminin, involved in the biological process. Surprisingly, we found that HPCs produced collagen and laminin. Moreover, silencing YB-1 promoted the expression of collagen I and laminin. ChIP sequencing and RNA sequencing identified *PDGFR-β* as a candidate target of YB-1, and the result was confirmed by ChIP-PCR, which indicated that YB-1 was able to directly bind to the *PDGFR-β* promoter. In addition, luciferase reporter assays demonstrated that YB-1 negatively modulated *PDGFR-β* promoter activity. Because PDGFR-*β* plays an extremely important role in liver fibrogenesis, we investigated its role in collagen expression in HPCs. We found that PDGF-*β* and PDGFR-*β* showed a similar trend with collagen I, and a PDGFR-*β* inhibitor and ERK1/2 inhibitor blunted collagen I expression after YB-1 knockdown.

Liver fibrosis is a wound-healing response accompanied by excessive deposition of extracellular matrix, which develops to cirrhosis if the damage persists. During the past decades, HSCs have been regarded as the major offenders contributing to liver fibrosis [12, 16]. In addition, other fibrogenic cells are reported to be involved, such as portal fibroblasts [17], fibrocytes [18], bone-marrow-derived cells [19], and cells derived from epithelial mesenchymal transition [20]. Moreover, HPCs express ECM-related genes [21]. Van Hul and colleagues reported that HPCs are embedded in ECM at all times, demonstrating that HPCs have the potential to synthesize ECM and cause hepatic fibrosis [22]. In this article, we provide more persuasive evidence to support the notion that HPCs also yield ECM, and this evidence is beneficial for completely uncover the mechanism of fibrogenesis. Due to the ability to differentiate into mature hepatocytes and cholangiocytes [23], HPCs are regarded as a reservoir to compensate for damaged hepatocytes or cholangiocytes, especially under perpetual injury conditions [24, 25]. When mice were fed a 50% choline-deficient, 0.15% ethionine-supplemented diet for 2 weeks, abundant HPCs, representing approximately 28% of the total liver cells, appeared around the periportal region of the liver. Based on these data, we proposed a new model in which HPCs contribute to liver fibrogenesis because of their vigorous proliferation rate and capacity to synthesize ECM.

YB-1 was originally identified as a transcription factor that regulates the expression of numerous genes, including *p53*, *MDR1*, *EGFR*, *cyclin A*, and *cyclin D1*. The biological activities in which YB-1 participates range from cell proliferation, differentiation and malignant cell transformation to cell adhesion. Merterns et al. reported that YB-1 directly bound to the promoter of the *collagen α1. (I)* gene and suppressed its promoter activity in human fibroblasts. Higashi and coworkers reported that YB-1 counter-repressed TGF-*β*-stimulated *collagen α2 (I)* transcription by interfering with Smad3 bound upstream of p300 in human dermal fibroblasts [26]. In addition, YB-1 was described to bind to the *Smad7* promoter and induce the expression of *Smad7* in rat HSCs, further inhibiting collagen expression [11]. These data suggest that YB-1 plays an inhibitory role in collagen expression, indicating that it may contribute to the fibrogenesis process. Thus, we sought to explore the effect of YB-1 on hepatic fibrogenesis in which activated HSCs play a predominant role. Herein, we identified that YB-1 knockdown with shYB-1 lentivirus vector promoted collagen I expression in HPCs through upregulation of PDGFR-*β*, which is different from the above mechanisms in which YB-1 modulates collagen I expression. In this study, collagen I was found to not be a target gene of YB-1 using ChIP sequencing (see Supplementary Table 1), which excludes the possibility that YB-1 binds to collagen I and regulates its expression.

For the first time, we identified *PDGFR-β* as a target gene of YB-1. To our knowledge, PDGF receptor signal transduction plays important roles in the embryo development, angiogenesis, and fibrotic diseases as well as in tumour development and migration [16, 27]. Elevated PDGF expression in cells expressing PDGFR has been demonstrated following both acute and chronic liver damage in experimental and human diseases. During liver fibrogenesis, PDGF-*β* has been recognized as the most potent mitogen for HSCs. In addition, PDGF-*β* is identified as a profibrogenic stimulus of HSCs using a transgenic mouse model [14]. However, our data demonstrate that the PDGF signalling network is involved in collagen synthesis in HPCs. To date, few articles have reported that PDGF signalling pathways are involved in the biological functions of HPCs. Only Lau et al. reported that the PDGFR-*β* inhibitor AG1296 suppressed the viability of a tumourigenic hepatic progenitor cell line (PIL2), which arises from p53 knockout in HPCs. In the present study, we show that HPCs express both PDGF-*β* and PDGFR-*β*, indicating that there may be an autocrine regulatory loop that modulates collagen synthesis in HPCs [28]. In addition, a serum-free culture system confirmed that HPCs secreted PDGF-*β*, and a PDGFR-*β* inhibitor inhibited the collagen production by HPCs. Further study demonstrated that ERK/p90RSK signalling mediates PDGFR-*β*-induced collagen expression in HPCs.

In conclusion, this study presents a novel mechanism in which YB-1 negatively modulates the expression of ECM in HPCs by negatively regulating the transcription of PDGFR-*β*. Disruption of the PDGFR-*β*/ERK/p90RSK signalling pathway represses the collagen expression in HPCs. The findings will help to uncover the mechanism of liver fibrogenesis, and interference with the regulatory axis may provide a new approach for treatment of liver fibrosis and cirrhosis.

## Supplementary Material

Supplementary Table 1: The information of binding sites that YB-1 binds to in hepatic progenitor cells, including the detailed locations and names of the closest genes. Supplementary Table 2: RNA sequencing screened 691 differentially expressed genes between YB-1knockdown HPCs and the scramble control using the following criteria: log2-fold change>0.585 or log2-fold change<-0.585, and p value<0.05.

## Figures and Tables

**Table 1:** Primer sequences for ChIP-PCR analysis.

Primer	Forward sequence	Reverse sequence	Product
PDGFR-*β*	CCAACTCAGGTGGCCTCTTA	TCACTGAACATCTGCCCTGT	82 bp
GAPDH	TGAGCTAGGACTGGTAAG	GTCCGTATTTATAGGAACCC	166 bp

**Table 2:** Primer sequences for RT-PCR analyses of gene expression.

Target	Forward primer	Reverse primer
YB-1	CAACAGGAATGACACCAAGG	TCAACAACATCAAACTCCACAG
PDGF-*β*	CAAGTGTGAGACAGTAGTG	ATGGGTGTGCTTAAACTTT
PDGFR-*β*	CAAAGGTGCTGGAGATGTTG	CAGTTGTTGCTGTCCGTGTT
Col1a1	CAAGAAGACATCCCTGAAG	GCAGATACAGATCAAGCATA
Col4a1	AGAGGAGGTGTATAGATAGC	GGAAGTCAGTCATTCAGTC
Laminin	CTTGGAACTGTTGGTAAGATA	ATCCTAATGAGCGGTTA
MMP13	GTGTGGAGTTATGATGATGT	ACTCTCACAATGCGATTAC
TIMP-1	ATCAACGAGACCACCTTA	CATATCCACAGAGGCTTTC
GAPDH	ACCACAGTCCATGCCATCAC	TCCACCACCCTGTTGCTGTA

**Table 3:** Antibodies used in Western blot (WB) analysis and immunofluorescence (IF).

Antibody	Dilution	Supplier	Product ID
YB-1	1 : 10,000 (WB), 1 : 1000 (IF)	Abcam	ab76149
p-YB-1 (ser102)	1 : 2000 (WB)	CST	2900
AFP	1 : 500 (IF)	Abcam	ab46799
Albumin	1 : 500 (IF)	Bethyl	A90-134A
CK19	1 : 500 (IF)	Abnova	MAB12076
CD133	1 : 100 (IF)	eBioscience	14-1331-80
CD90/Thy1	1 : 500 (IF)	Novus	NB100-65543
*α*-SMA	1 : 500 (IF)	Abcam	ab7817
Collagen I	1 : 1000 (WB), 1 : 200(IF)	Abcam	6308
PDGF-*β*	1 : 2000 (WB)	Abcam	ab178409
PDGFR-*β*	1 : 2000 (WB)	Abcam	ab32570
p-Stat3	1 : 1000 (WB)	CST	9145
ERK1/2	1 : 1000 (WB)	CST	4695
p90 RSK (ser380)	1 : 1000 (WB)	CST	9341
PI3K p85	1 : 1000 (WB)	R&D	MAB2998
GAPDH	1 : 10,000 (WB)	Abcam	ab8245

**Table 4:** Transcripts upregulated in ECM-receptor interaction.

Gene name	Gene	Fold change	YB-1 KD-RPKM	Scramble-RPKM
Collagen, type III, alpha 1	Col3a1	4.58	1.89125	0.412824
Collagen, type V, alpha 2	Col5a2	3.40	4.68447	1.37872
Thrombospondin 2	Thbs2	2.15	0.414252	0.193934
Reelin	Reln	1.89	0.916106	0.48511
Thrombospondin 1	Thbs1	1.79	93.503	52.3547
Collagen, type I, alpha 2	Col1a2	1.78	5.134	2.87674
Collagen, type IV, alpha 2	Col4a2	1.69	119.857	70.8253
Collagen, type IV, alpha 1	Col4a1	1.63	202.428	123.942
Integrin beta 6	Itgb6	1.61	6.48106	4.03229

## Abbreviations

ChIP: Chromatin immunoprecipitation
ECM: Extracellular matrix
EGF: Epidermal growth factor receptor
ERK: Extracellular signal-regulated kinase
HPCs: Hepatic progenitor cells
HSCs: Hepatic stellate cells
PDGF: Platelet-derived growth factor
PDGFR: Platelet-derived growth factor receptor
RSK: Ribosomal S6 kinase
RT-PCR: Reverse transcriptase polymerase chain reaction
TGF: Transforming growth factor
YB-1: Y-box protein-1.

## Additional Points

*Highlights.* (1) Hepatic progenitor cells synthesize extracellular matrix. (2) YB-1 directly binds to the *PDGFR-β* promoter and negatively regulates PDGFR-*β* transcription. (3) YB-1 knockdown enhances collagen I expression via PDGFR-*β*/ERK/p90RSK signalling.
